# Psychological impact of COVID-19 in the Swedish population: Depression, anxiety, and insomnia and their associations to risk and vulnerability factors

**DOI:** 10.1192/j.eurpsy.2020.81

**Published:** 2020-08-26

**Authors:** Lance M. McCracken, Farzaneh Badinlou, Monica Buhrman, Karin C. Brocki

**Affiliations:** 1 Division for Clinical Psychology, Department of Psychology, Uppsala University, Uppsala, Sweden; 2 Division for Emotion Psychology, Department of Psychology, Uppsala University, Uppsala, Sweden.

**Keywords:** Anxiety, COVID-19, depression, insomnia, mental health, risk and vulnerability factors

## Abstract

**Background:**

The 2019 coronavirus disease (COVID-19) pandemic, with its associated restrictions on daily life, is like a perfect storm for poor mental health and wellbeing. The purpose of this study was therefore to examine the impacts of COVID-19 on mental health and wellbeing during the ongoing pandemic in Sweden.

**Method:**

Standardized measures of depression, anxiety, and insomnia as well as measures of risk and vulnerability factors known to be associated with poor mental health outcomes were administered through a national, online, cross-sectional survey (*n* = 1,212; mean age 36.1 years; 73% women).

**Result:**

Our findings show significant levels of depression, anxiety, and insomnia in Sweden, at rates of 30%, 24.2%, and 38%, respectively. The strongest predictors of these outcomes included poor self-rated overall health and a history of mental health problems. The presence of COVID-19 symptoms and specific health and financial worries related to the pandemic also appeared important.

**Conclusions:**

The impacts of COVID-19 on mental health in Sweden are comparable to impacts shown in previous studies in Italy and China. Importantly, the pandemic seems to impose most on the mental health of those already burdened with the impacts of mental health problems. These results provide a basis for providing more support for vulnerable groups, and for developing psychological interventions suited to the ongoing pandemic and for similar events in the future.

## Background

The world has changed dramatically in the wake of the 2019 coronavirus disease (COVID-19). This condition first appeared in December of 2019, and began to spread worldwide. It was deemed a Public Health Emergency by the World Health Organization (WHO) in January 2020, and was pronounced a pandemic in March [1,2]. By the last week of May, there were already 5.93 million confirmed cased worldwide and over 367,000 related deaths [3]. The public health, social, and economic impacts of COVID-19 are only now being calculated, and appear enormous. There is concern that this pandemic could lead to significant impacts on mental health and wellbeing [4–6], but these impacts are currently unknown. Assessing these will be important as the world navigates a process of recovery and plans for future disease outbreaks.

The COVID-19 virus presents a highly communicable threat to health, and a concerning mortality rate, where some of us are more at risk due to older age, gender, and underlying disease [7]. Due to ease of transmission and high mortality, authorities around the world have implemented social distancing and washing-hands/hygienic guidelines, stay-at-home orders, quarantine, and closures/lock down of schools, leisure activities, businesses, transport systems, and borders. These types of restrictions create additional social and economic burden, and seem very likely to carry additional impacts on mental health and wellbeing, as people isolate from others, work from home or lose jobs, lose wages, incur debt, reduce physical activity and exercise, and possibly reside in small spaces with others, unable to get away [5].

To this point, published results on psychological impacts of COVID-19 have emerged from populations surveys conducted in China [8–10], Italy [11], and the United States [12]. In the largest of these studies (*N* = 2,766), conducted in Italy, respondents provided data on depression, anxiety, and stress, and the percentage of respondents at the high or extremely high level of these mental health outcomes was 32.8, 18.7, and 27.2%, respectively [11]. These figures can be compared to results from a study in China (*N* = 1,210), using the same measures, where the percentages of respondents scoring moderate to severe on depression 16.5%, anxiety 28.8%, and stress 8.1% [10]. Another study in China (*N* = 1,074), this time using different measures, showed 26.9% moderate to severe depression and 18.9% moderate to severe anxiety [8].

Variation in prevalence of mental health problems during the pandemic across countries is naturally confounded by nonpandemic factors specific to each country such as culture, political policies, and economic situation, and also time since onset of the pandemic. Nevertheless, when comparing national statistics for mental health prevalence in each country before the pandemic with the findings from the national surveys conducted during the pandemic it is evident that the frequency of mental health problems is substantially higher in the period of COVID-19 [8,11,13], which certainly points to the pandemic as a plausible cause. In fact, between one in six and one in three people report a significant level of depression or anxiety particularly during this time of COVID-19. Also important is that while the rates of these outcomes may vary across countries and time, essentially all of the rates raise concern.

In Sweden, the confirmed cases in the last week of May were  38,664 and related deaths were 4,633. For Sweden the crisis had arrived and remained more or less at a peak during this time [3] and the overall mortality rate was high, especially in residential care setting for older people. Swedish authorities did not force people to stay at home, impose strict social-distancing policies, or close borders, and many businesses and childcare facilities stayed open [14]. Instead, people were advised to work from home, when possible, meetings of more than 50 people were banned, businesses and higher education voluntarily turned to video conferencing and reduced non-essential travel [15]. There is no doubt that life changed in Sweden, but perhaps not to the extent as in other countries, particularly around Europe, China, and the United States. It has been shown that being under a stay at home order is associated with greater health anxiety, financial worry, and loneliness [12]. Whether this effect has been mitigated in Sweden is not known.

The purpose of this study was to examine if the prevalence of mental health problems during the COVID-19 pandemic exceed estimated nonpandemic prevalence rates for depression, 10.8%, anxiety, 14.7%, and insomnia,  7-10%, in Sweden [16,17,18] through a national web survey. Here measures of depression, anxiety, and insomnia were administered, depression and anxiety as they are common dimensions of mental health, and insomnia because it is sensitive to stressful events and an important predictor of emotional and physical health [19,20]. Of course, the overlap between insomnia, anxiety, and depression is well known [21] and this can be further investigated here. Studies conducted during previous infectious disease outbreaks, such as severe acute respiratory syndrome (SARS) and Middle East Respiratory Syndrome (MERS) have shown that anxiety, depression, and insomnia were the most prevalent mental health problems during these disease outbreaks [22–24]. In the present study, we assess associations between these mental health problems and risk factors previously associated with psychological impacts in the context of a flu pandemics [25], such as demographic characteristics, infection exposure, symptoms, and specific worries related to COVID-19. We also examine the associations between history of mental health problems and the outcome variables as previous study showed that the psychological impacts of COVID-19 are more prominent in people with pre-existing mental health problems [26]. The study findings could provide the means for addressing the immediate aftermath of the current pandemic and plan for similar health threats in the future.

## Methods

### Participant and procedure

A population-based cross-sectional study was conducted in order to explore the Swedish community’s reactions to the COVID-19 pandemic. Between May 14 and June 11, 2020, adult participants (≥18 years) were recruited through advertisements about our study on social media platforms (Facebook and Twitter) and Uppsala University and Uppsala University hospital homepages. The survey was administered using the online survey platform Research Electronic Data Capture (REDCap) tool as hosted by Uppsala University. REDCap is a secure web application for building, managing, and supporting online surveys and data capture for research studies. The final sample consisted of 1,212 participants (73% women) representing all 21 counties in Sweden (see Table 1 for demographics). Online informed consent was obtained from all participants. The participants were fully informed about the aims of the study, that their participation was voluntary, and that they were free to discontinue participation at any time. The participants did not receive any compensation for taking part in the study. The study was approved by the Swedish National Ethical Board (dnr 2020/1910).

**Table 1.** Demographic characteristics and physical and psychological status of the participants (*N* = 1212).

Characteristics	*n*	%
Gender		
Female	895	73.8
Male	317	26.2
Working status		
Unemployed/unpaid work	45	3.7
Working part time	162	13.4
Working full time	529	43.6
Parental leave	22	1.8
Sick leave	29	2.4
Retired	72	5.9
Student	353	29.1
Education		
Presecondary	43	3.5
Secondary	353	29.1
Postsecondary	704	58.1
Graduate studies	112	9.2
Income^a^		
Lower middle-income	534	44
Middle-income	339	28
Upper middle-income	248	20.5
High-income	91	7.5
Marital status		
Single	461	38
Married/ in a relationship	677	55.9
Separated/divorced	58	4.8
Widowed	16	1.3
Place of residence		
In the Countryside	201	16.6
Villa in the suburbs	231	19.1
Apartment in the suburbs	207	17.1
In the city	573	47.3
Number of children under 18 years		
No child	843	69.6
1	126	10.4
2	146	12
3 and more	49	4.1
Household size		
1	298	24.6
2	402	33.2
3	200	16.5
4	219	18.1
5 and more	93	7.7
Country of birth		
Sweden	937	77.3
Scandinavian countries	34	2.8
Other European countries	115	9.5
Latin American countries	22	1.8
North American countries	22	1.8
Asian countries	68	5.6
African countries	12	1
Oceania countries	2	0.2
Physical risk factors		
No physical risk factor	851	70.2
1	251	20.7
2	75	6.2
3	21	1.7
More than 3	14	1.3
Households with other person at risk		
Yes	301	24.8
No	911	75.2
History of mental health problems		
Yes	461	38
No	751	62

a Lower middle-income: 0–200,000 SEK/year (before tax); middle-income: 200,000–400,000 SEK/year (before tax); upper middle-income: 400,000–600,000 SEK/year (before tax); high-income: over 600,000 SEK/year (before tax).

The questionnaires in this study were designed and guided by previous pandemic studies [25] on public psychological reactions, guidelines of WHO and the public health agency of Sweden. The data gathered consisted of basic demographic information, risk and vulnerability factors, and COVID-19 status. In addition, standardized and validated questionnaires were administered with the aim to measure mental health in terms of depression, anxiety, and sleep.

### Measures

#### Demographic variables

Information about age, gender, place of residence, working status, education, yearly income, marital status, number of children under 18-years-old, household size, and country of birth were collected.

#### Risk and vulnerability

We measured the presence of physical health risk for adverse outcomes from COVID-19 with 10 items developed for the purpose of this study according to guidelines of WHO [27] and the Public Health Agency of Sweden [28]. The risk factors included age over 70 years, high blood pressure, angina, stroke, heart disease, diabetes, cancer, smoking, respiratory diseases, and impaired immune system. Participants were also asked whether somebody in their household had any of these same risk factors. Psychological risk was assessed by asking about history of mental health problems and previous psychiatric diagnosis (yes or no). Current overall perceived health was measured with a single item, in which respondents were asked to rate their current health state on a 5-point scale from 1 (very poor) to 5 (very good).

#### COVID-19 status

Current physical symptoms of COVID-19 were assessed with 17 items, including 16 items from previous flu pandemic study [25], fever, chills, headache, aches or pain in body, fatigue, diarrhea, sore throat, runny or blocked nose, sneezing, loss of appetite, difficulty sleeping, coughing, sinus problems, cough, nausea, shortness of breath, stomach ache, and a final item, loss of smell and taste in the past seven days, based on findings from COVID-19 specifically. Responses were yes or no. Individuals further indicated if they had been tested for COVID-19 and if they had been diagnosed with COVID-19. COVID-19-related worry was assessed with four items asking about worry about their own health, their families’ and friends’ health, their own financial circumstances, and financial circumstances in Sweden and world on a 5-point scale from 1 (Not at all worried) to 5 (Extremely worried).

#### Depression

The Patient Health Questionnaire (PHQ-9) is a 9-item depression scale based on the diagnostic criteria of DSM IV. The PHQ-9 has a dual purpose to establish provisional depressive disorder diagnoses as well as to grade depressive symptom severity. Item 9 assesses suicidal thinking. An additional item at the end of the PHQ-9 is a global rating of functional impairment [29]. The PHQ-9 score ranges from 0 to 27. PHQ-9 shows adequate reliability and convergent/discriminant validity [30]. The suggested cutoff points for mild, moderate, moderately severe, and severe depression are 5, 10, 15, and 20, respectively [31]. The internal consistency for the PHQ-9 based on the current sample was *α* = 0.89.

#### Anxiety

The General Anxiety Disorder−7 (GAD-7) consists of seven items and was initially created as a screening tool for General Anxiety Disorder in primary care settings [32]. It is used as a measure of general anxiety symptoms across various settings and populations [33–35]. Scores on the GAD-7 range from 0 to 21 and a cutoff score of 10 has been identified as the optimal point for sensitivity (89%) and specificity (82%) [36]. GAD-7 has demonstrated strong psychometric properties in the general population [32–34]. Normative data provided for the general population showed that approximately 5% of subjects had GAD-7 scores of 10 or greater, and 1% had GAD-7 scores of 15 or greater [35]. The internal consistency of the GAD-7 based on the current sample was *α* = 0.90.

#### Insomnia

The Insomnia Severity Index (ISI) is a brief instrument consisting of seven items and assesses the nature, severity, and impact of insomnia. The dimensions evaluated include severity of sleep onset, sleep maintenance, early morning awakening problems, sleep dissatisfaction, interference of sleep difficulties with daytime functioning, noticeability of sleep problems by others, and distress caused by the sleep difficulties. The total score ranges from 0 to 28, and is interpreted as follows: absence of insomnia (0–7); sub-threshold insomnia (8–14); moderate insomnia (15–21); and severe insomnia (22–28) [37]. The ISI has been evaluated in a population-based sample and has adequate psychometric properties. It is suggested a cutoff score of 10 (86.1% sensitivity and 87.7% specificity) for detecting insomnia in a general population [38]. The internal consistency for the ISI-7 in the present study was *α* = 0.90.

### Statistical analyses

Data were analyzed with SPSS version 23.0. First, a summary of the potential risk factors ( demographic variables, risk and vulnerability factors, COVID-19 status) and the outcome variables of mental health (depression, anxiety, and insomnia) is presented in Tables 1 and 2. Then, associations between the potential risk factors and the outcome variables of mental health were calculated with Pearson’s correlation or with analysis of variance (ANOVA). Finally, three separate hierarchical multiple regression analyses were performed with the PHQ, GAD, and ISI as outcome variables, respectively, to examine the independent contribution of risk factors that were significantly related to mental health problems in the correlation analyses. The significant factors were categorized into four blocks entered in the following order: demographic variables, risk and vulnerability, exposure to COVID-19, and worries related to COVID-19, in all three regression models.

**Table 2.** Prevalence of mental health variables.

Mental health	Mean (SD)	Score	*n* (%)
PHQ (0–27)	7.28 (6.3)		
Minimal or no depression		0–4	513 (42.3)
Mild depression		5–9	336 (27.7)
Moderate depression		10–14	190 (15.7)
Moderately severe depression		15–19	98 (8.1)
Severe depression		20–27	75 (6.2)
GAD (0–21)	6.05 (5.6)		
No anxiety		0–4	599 (49.4)
Mild anxiety		5–9	320 (26.4)
Moderate anxiety		10–14	165(13.6)
Severe anxiety		15–21	128 (10.6)
ISI (0–28)	8.32 (6.3)		
Absence of insomnia		0–7	620 (51.2)
Sub-threshold insomnia		8–14	359 (30.1)
Moderate insomnia		15–21	178 (14.7)
Severe insomnia		22–28	37 (3.1)

Abbreviations: GAD, General Anxiety Disorder; ISI, Insomnia Severity Index; PHQ, Patient Health Questionnaire; SD, standard deviation.

## Results

### Descriptive statistics for risk factors and mental health variables

Demographic information about the participants is presented in Table 1. The participants’ age ranged between 18 and 88 years old, (*M* = 36.1, standard deviation [SD] ± 15.2 years old; *women*: *M* = 36.6, SD ± 15.4 years old; *men*: M = 34.8, SD ± 14.7 years old). Among them 46.9% (*n* = 568) were aged 18–30, 19.1% (*n* = 232) were aged 31–40, 14.8% (*n* = 179) were aged 41–50, 10.9% (*n* = 132) were aged 51–60, and 8.3% (*n* = 101) were aged 61–88. The majority of the participants had no physical risk factors 70.2% (*n* = 851), but among the risks reported, smoking 12.4% (*n* = 150), high blood pressure 9.2% (*n* = 112), respiratory diseases 7.5% (*n* = 91), and diabetes 3.7% (*n* = 45) were the most prevalent. Most of the participants reported their current health status as being average or good 74.5% (*n* = 930). Further, 21.6% (*n* = 262) of the participants reported that they had received a psychiatric diagnosis, which is consistent with lifetime prevalence of mental disorders [39].

Forty-six percent of the participants reported between one and four symptoms of COVID-19 in the past seven days, with fatigue/tiredness/low energy (*n* = 759, 62.9%), headache/migraine (*n* = 571, 47.1%), runny/blocked nose/snot (*n* = 492, 40.6%), and aches/pain in muscles/bone/joints (*n* = 398, 32.8%) being the most prevalent reported symptoms among the participants. Five percent of the participants had been tested for COVID-19 and 2% had been diagnosed with COVID-19. The COVID-19 related worry items, each rated 1 to 5, yielded means of 2.29 (SD: 1.14) for own health, 3.38 (1.19) for health of family and friends, 2.34 (1.36) for personal finances, and 3.34 (1.15) for Swedish and world finances.

Table 2 presents the prevalence rates of depressive symptoms (PHQ), anxiety (GAD), and insomnia (ISI). Of all participants 30% (*n* = 363), 24.2% (*n* = 329), and 38% (*n* = 457) reached the cutoff for clinical depression, anxiety, and insomnia (score ≥ 10), respectively. The data from the suicidal thinking item from the PHQ-9 showed that a significant number people, 14.9%, (*n* = 180) endorsed “Thoughts that you would be better off dead, or of hurting yourself” in the past two weeks, and 5.4% had these thoughts more than half the time or nearly every day. Further, on the PHQ-9 65.2% individuals reported a significant impact on their daily functioning from their symptoms of depression.

### Relations between risk factors and mental health variables

As can be seen in Table 3, age, number of children, and general health status were negatively related to all three of the mental health variables, whereas physical risk factors, other person at riskin the household, history of mental health problems, COVID-19 symptoms, and COVID-19-related worry were positively related to mental health variables. The intercorrelations between depression and anxiety, depression and insomnia, and anxiety and insomnia were 0.84, 0.68, and 0.67, respectively, (*p* < 0.01).

**Table 3.** Pearson correlations between demographic variables, risk and vulnerability factors, COVID-19 status and PHQ, GAD, and ISI.

Mental health variables	PHQ	GAD	ISI
Age	−0.297**	−0.305**	−0.156**
Gender	−0.05	−0.035	−0.028
Number of children under 18	−0.142**	−0.134**	−0.094**
Household size	0.009	0.013	0.017
Physical risk factors	0.055	0.064*	0.084**
Household with other person at risk	0.106**	0.126**	0.085**
History of mental health problems	0.534**	0.547**	0.419**
General health status	−0.462**	−0.385**	−0.422**
COVID-19 symptoms	0.420**	0.371**	0.411**
Tested for COVID-19	−0.029	0.001	0.008
Diagnosed with COVID-19	0.009	0.002	−0.002
Worry about own health	0.252**	0.343**	0.256**
Worry about the health of family and friends	0.355**	0.443**	0.311**
Worry about own economy	0.443**	0.476**	0.351**
Worry about economy in Sweden and world economy	0.175**	0.211**	0.134**

Abbreviations: COVID-19, 2019 corona virus disease; GAD, General Anxiety Disorder; ISI, Insomnia Severity Index; PHQ, Patient Health Questionnaire.

* *p* < 0.05;

** *p* < 0.01.

Table 4 presents the results from the ANOVA and shows that the effects of marital status, work status, education, income, and place of residence on mental health variables were significant. Post hoc comparisons using Tukey HSD test showed that high levels of PHQ, GAD, and ISI scores were significantly associated with single material status, unemployment, working part time, sick leave and student work status, lower education, lower income, and living in an apartment in the suburbs.

**Table 4.** Effects of demographic characters on mental health variables, means (*M*), standard deviations (SD) and *F* statistics.

Variables	Depression, *M* (SD)	Anxiety, *M* (SD)	Insomnia *M*, (SD)
Marital status			
Single	9.13 (6.6)	7.3 (5.7)	9.4 (6.2)
Married/ in a relationship	6.08 (5.8)	5.2 (5.2)	7.5 (6.2)
Separated/divorced	6.8 (5.4)	5.3 (5.1)	8.8 (6.7)
Widowed	5.8 (6.9)	4.7 (7.5)	7.5 (6.3)
*F*	22.66** 1 > 2; 1 > 3	15.02** 1 > 2; 1 > 3	7.82* 1 > 2
Work status			
Unemployed/unpaid work	11.15 (8.05)	8.8 (6.9)	12.3 (6.09)
Working part time	7.6 (6.7)	6.6 (5.7)	8.6 (6.7)
Working full time	6.03 (5.4)	5.06 (4.9)	7.6 (5.9)
Parental leave	5.8 (5.1)	5.1 (4.7)	6.8 (5.5)
Sick leave	11.8 (7.2)	9.3 (6.3)	10.9 (6.7)
Retired	4.01 (4.7)	3.2 (4.8)	6.9 (7.02)
Student	8.9 (6.5)	7.2 (5.5)	8.9 (6.3)
*F*	17.22** 1 > 3; 1 > 4; 1 > 6; 2 > 6; 5 > 3; 5 > 4; 5 > 6; 7 > 3 7 > 6	13.59** 1 > 3; 1 > 6; 2 > 3; 5 > 3; 5 > 6; 7 > 3 7 > 6	6.05** 1 > 3; 1 > 4; 1 > 6
Education			
Presecondary education	8.8 (6.6)	7.5 (6.5)	9.9 (6.9)
Secondary education	9.6 (6.8)	8.1 (5.8)	9.7 (6.6)
Postsecondary education	6.4 (5.8)	5.3 (5.1)	7.9 (6.1)
Graduate education	4.1 (4.7)	3.7 (4.4)	6.08 (5.2)
*F*	32.28** 1 > 4; 2 > 3; 2 > 4	29.73** 1 > 3; 1 > 4; 2 > 3; 2 > 4	12.38** 1 > 4; 2 > 3; 2 > 4
Income			
Lower middle-income	9.08 (6.7)	7.6 (5.8)	9.4 (6.6)
Middle-income	6.8 (5.7)	5.8 (5.2)	8.4 (6.1)
Upper middle-income	5.3 (5.5)	4.1 (4.6)	6.5 (5.6)
High-income	3.7 (4.7)	2.9 (4.06)	6.3 (5.8)
*F*	35.20** 1 > 2; 1 > 3; 1 > 4; 2 > 3; 2 > 4	37.43** 1 > 2; 1 > 3; 1 > 4; 2 > 3; 2 > 4	15.23** 1 > 2; 1 > 3; 1 > 4; 2 > 3; 2 > 4
Place of residence			
In the countryside	7.1 (6.5)	5.7 (5.6)	8.4 (6.7)
Villa in the suburbs	6.2 (6.3)	5.2 (5.3)	7.8 (6.1)
Apartment in the suburbs	8.8 (6.8)	7.7 (5.8)	9.3 (6.5)
In the city	7.1 (5.9)	5.8 (5.3)	8.1 (6.1)
*F*	6.43** 1 > 2; 1 > 3; 1 > 4	8.64** 1 > 2; 1 > 3; 1 > 4	2.64* 1 > 2

* *p* < 0.05;

** *p* < 0.01.

### Independent contributions of risk and vulnerability factors to explained variance in mental health problems

Results from the three separate hierarchical regression models are presented in Table 5. Although all of the four blocks contributed significant, unique, explained variance in mental health problems, the risk and vulnerability factors and particularly history of mental health problems contributed with the largest part of the variance in each model. Further, the presence of COVID-19 symptoms, as well as worry about one’s personal finances, also stood out as an important predictor in the models for all three mental health problems (see Table 5).

**Table 5.** Hierarchical multiple regression analyses of demographic, risk and vulnerability factors, exposure to COVID-19, and COVID-19 related worry and mental health variables.

Independent variables	Dependent variables							
	Depression			Anxiety			Insomnia	
	Δ*R^2^*	*β*		Δ*R^2^*	*β*		Δ*R^2^*	*β*
Block 1: Demographic variables	0.138***			0.138***			0.055***	
Age		−0.95**			−0.130***			−0.007
Marital status^a^		−0.084**			−0.029			−0.052
Children under 18		−0.043			−0.036			−0.036
Work status^b^		−0.042			−0.007			0.011
Education^c^		−0.046			−0.045			−0.019
Income^d^		0.014			−0.012			−0.040
Place of residence^e^		0.004			0.020			0.002
Block 2: Risk and vulnerability factors	0.269***			0.257***			0.187***	
Physical risk factors		−0.001			0.015			0.000
General health status^f^		−0.154***			−0.082**			−0.105***
History of mental health problems		0.307***			0.327***			0.237***
Household include other person at risk		0.043*			0.062**			0.032
Block 3: Exposure to COVID-19	0.05***			0.04***			0.069***	
COVID-19 symptoms		0.217***			0.173***			0.264***
Block 4: COVID-19 related worries	0.043***			0.078***			0.028***	
Worry about own health		0.039			0.106***			0.061*
Worry about family and friends’ health		0.065**			0.120***			0.061*
Worry about own economy		0.163***			0.165***			0.118***
Worry about global economy		0.055*			0.06**			0.028

a Marital status categorized into being single versus not being single (i.e., married/in a relationship, separated/divorced, and widowed).

b Work status categorized into unstable economy (i.e., unemployment, sick leave, working part-time and student) versus stable economy (i.e., working full time, parental leave, and retired).

c Education as categorized into lower-level education (i.e., presecondary and secondary education) versus higher level education (i.e., undergraduate and graduate education).

d Income as categorized into lower level income (i.e., lower middle-income and middle income) versus higher level income (i.e., upper middle-income and high-income).

e Place of residence as categorized into living in an apartment versus not living in an apartment (i.e., living in the countryside, villa in the suburbs, and in the city).

f General health status as categorized into good versus poor health status.

* *p* < .05;

** *p* < .01;

*** *p* < .001.

## Discussion

The purpose of this survey was to map the effects on mental health and wellbeing of COVID-19 in the Swedish population and to explore whether they exceed the typical nonpandemic prevalence of depression, anxiety and insomnia. It was conducted 2.5 months after the first appearance of COVID-19 on 31st January. It shows significant rates of significant depression (30%), anxiety (24.2%), and insomnia (38%) in Sweden. In correlation analyses, factors significantly positively associated with these measures of mental health included having a person in the household at risk for adverse outcomes from COVID-19, living in an apartment in a suburban setting, being unemployed, working part-time, being on sick leave or student, being single, having a history of a mental health problem, having current symptoms of COVID-19, and worries about health, family, or the economy. Factors significantly negatively correlated with these measures included age, education, income, number of young children in the home, and self-rated general health status. Importantly, the history of a mental health problem stood out as the strongest predictor of all the mental health outcomes assessed. In multivariate models, generally, background vulnerability factors, including self-rated poor overall health, and a history of mental health problems contributed the most to the explained variance in depression, anxiety, and insomnia. At the same time, the presence of COVID-19 symptoms and specific worries related to this condition also contributed with significant explained variance.

Looking at depression, anxiety, and insomnia in combinations with each other reveals a further clarifying perspective on impact. The number of people who meet criteria for a significant problem in any of the mentioned conditions is 45.6%, leaving 54.4% meeting criteria for no condition. Naturally, it is also observed here that these conditions are highly comorbid. In fact, the proportion of people meeting criteria for significant symptoms in all three conditions is 16.9%, which is the same as the proportion who meet criteria for any single one, 17.0%, and both of these proportions are greater than the number who meet criteria for any two, 11.6%. A meaningful number of the people surveyed describe problems at a severe level, including 6.2% (*n* = 75) of the sample reporting severe depression, 10.6% (*n* = 128), severe anxiety, and 3.1% (*n* = 37) severe insomnia.

Furthermore, 180 people, or 14.9% of the total sample, reported having thoughts of suicide in the past two weeks, including 5.5% reporting having these thoughts more than half the time or nearly every day. Importantly, out of the positive cases, 28.6% report no prior mental health problems, which may mean that approximately 1/3 are experiencing clinically significant levels of depression, anxiety and/or insomnia for the first time during the pandemic.

Results here addressing the possible psychological impacts of COVID-19 in Sweden appear to show that, overall, these impacts are no worse, and no better, than impacts shown in previous studies in Italy [12] or China [9,10]. Differences in sampling and recruitment methods, and in the measures used, in addition to the time frame in pandemic terms, make precise comparison difficult. However, consistently, in each country and at each time point, results suggest that for any one mental health and wellbeing variable assessed, significant proportions of people appear adversely affected, roughly between one in six and one in three people, in any one of the variables assessed. These rates of likely significant depression, anxiety, and insomnia identified here at 30.0, 24.2, and 38% are substantially higher than the rates of 10.8, 14.7, and 7-10% found in Swedish general population surveys, during nonpandemic times, using the same measures for depression and anxiety, but a different measure for insomnia [18,39,40]. We note that in this previous study they used a cutoff of 8 for the GAD-7 unlike the cutoff of 10 used here, making this difference even more substantial. Our rates can also be compared with a national survey on mental health conducted by the Public Health Agency of Sweden in 2018 (*n* = 16631; age 16–84 years) [41]. In this survey 17% of participants reported impaired mental health and wellbeing, 7% reported having high levels of anxiety and worry and 7% significant sleep problems. Although the national survey used a different measure, the General Health Questionnaire (GHQ-12), assessing psychological wellbeing and psychological reactions to life stressors, rather than mental illness, the much higher rates for poor mental health in our study are clear, and in line with previous studies [26].

Factors that appeared unassociated with mental health and wellbeing included gender, household size, having been tested for, or diagnosed with, COVID-19, the latter two due to extremely low rates of these. This could be explained by the fact that testing of COVID-19 has been limited in Sweden during the period of the data collection for the present study.

On the whole, background demographic factor did not account for the largest share of variance in the multivariate analyses, despite their entry as the first block of potential predictors. Among these, only age emerged as consistently, significantly, and uniquely negatively associated with mental health and wellbeing. This means that younger persons seem to be more affected than older individuals, this finding is in line with a pervious study that stated that people in the younger age groups experienced higher level of psychological distress during the COVID-19 outbreak [12,13,42]. It may be that young people have less financial security or have less social stability, being in a transition phase of their lives. Further, this finding also corroborates the general (nonpandemic) finding that younger people suffer from mental health problems to a greater extent than older people [43]. From this perspective, it is quite reasonable that younger people may be more vulnerable to the potential impact of the pandemic on poor mental health and wellbeing.

Once again, the block of potential predictors that we refer to as background vulnerability factors consistently accounted for the most variance, with change *Δ* *R* ^2^ values demonstrating 26.9, 25.7, and 18.7% variance accounted for in depression, anxiety, and insomnia, respectively. This is in a context of *R* ^2^ values reflecting total variance explained at 50, 51, and 33%, again, respectively. The presence of COVID-19 symptoms themselves consistently appeared significant in the models, not surprisingly, although the variance accounted for was modest, at 5.0, 4.0, and 6.9%. And finally, we also examined specific worries in the form of worries about own health, family or friends’ health, personal finances, and world economy. As a block these consistently accounted for significant variance in all models, at 4.3, 7.8, and 2.8%. Among the worries, worry over one’s own personal finances was clearly and consistently the one most linked to depression, anxiety, and insomnia. This finding relates to recent results by Taylor et al. [44] demonstrating a strong association between fear of perceived threat of COVID‐19 and COVID‐19‐related socioeconomic consequences.

The results of this survey should be seen in the context of who the respondents were. Most of the respondents were women, less than 40 years old, and born in Sweden. Most were working full time or were students. Most had no underlying condition that would place them at particular risk for a poor outcome from COVID-19 exposure. Where underlying conditions conveying risks were reported, these were most often smoking, hypertension, or respiratory disease.

The study was planned and executed in a shorter than usual time frame, to attempt to capture the impact of extraordinary and quickly developing events. Naturally, there are significant limitations in the methods employed here. While a relatively large sample was obtained, the recruitment methods here are selective, relying as they do on social media channels and other local websites. It may be that social media particularly attracts people who are distressed, and looking for answers or for support [45]. This could bias the data. Although we are encouraged by the wide participation across regions of Sweden, representativeness of our sample, and generality, cannot be assured. Instead, it is hoped that other research groups will also have conducted similar studies, including in Sweden, so that a wider pattern and potential reliability can be seen. Other limitations include the cross-sectional design and the fallible nature of self-report measures in a setting like this. There will be inaccuracies and uncertainty around our estimates of depression, anxiety, and insomnia. Although we had research guidance on survey design in the context of flu pandemics [25], we have no way to know whether the potential predictors chosen have missed others that could have been more important. In this sense our models need to be seen as tentative.

Practical recommendations suggested from the results here would be to provide greater support for those most vulnerable to adverse impacts on mental health and wellbeing. This includes those with past or current mental health problems or those who regard themselves as having relatively poorer overall health. On a societal level there is an argument that seems obvious. If we want better population mental health and wellbeing during such events as a flu pandemic, we need to protect people from threats to their physical and mental health, and probably also protect them against threats to their livelihoods. It should be noted that in the unadjusted analyses the worries about pandemic impacts, including financial ones, were among the potential predictors most strongly associated with the outcome assessed. Further studies will be needed to better pinpoint potential public health or clinical therapeutic targets for intervention and then to develop and test interventions, including efficacy, cost-effectiveness, and implementation.

## Conclusion

At what appears to have been the height of the COVID-19 pandemic in Sweden, so far, 45.6% of Swedish residents responding to an online survey reported symptoms reflecting significant problems in one or more areas of their mental health, including depression, anxiety, or insomnia. Also, having multiple problems was the norm—people were almost twice as likely to have more than one of these problems than they were to have just one. Each of the problems examined here, generally speaking, appeared most often in those who are most vulnerable in their health and socioeconomic circumstances. In multivariate models including all significantly associated factors from bivariate analyses, self-rated health and mental health history appeared consistently among the most strongly associated correlates of depression, anxiety, and insomnia. These were, arguably, more important than preexisting physical health conditions linked to COVID-19 risk or the actual symptoms of COVID-19 themselves. One might say that the pandemic imposes most particularly on the mental health of those already burdened with the impacts of mental health problems. Worries related to impacts of COVID-19 also figured importantly in these models, and among these the greatest impact may be from worries over personal or household finance.

## Data Availability

We make our resources publicly available.
